# Association between the *BRCA2* rs144848 polymorphism and cancer susceptibility: a meta-analysis

**DOI:** 10.18632/oncotarget.16242

**Published:** 2017-03-15

**Authors:** Qiuyan Li, Rongwei Guan, Yuandong Qiao, Chang Liu, Ning He, Xuelong Zhang, Xueyuan Jia, Haiming Sun, Jingcui Yu, Lidan Xu

**Affiliations:** ^1^ Laboratory of Medical Genetics, Harbin Medical University, Harbin, People's Republic of China; ^2^ Department of Clinical Laboratory, Qiqihar Traditional Chinese Medicine Hospital, Qiqihar, People's Republic of China; ^3^ The Second Affiliated Hospital, Harbin Medical University, Harbin, People's Republic of China

**Keywords:** meta-analysis, BRCA2, cancer, polymorphism, susceptibility

## Abstract

The *BRCA2* gene plays an important role in cancer carcinogenesis, and polymorphisms in this gene have been associated with cancer risk. The *BRCA2* rs144848 polymorphism has been associated with several cancers, but results have been inconsistent. In the present study, a meta-analysis was performed to assess the association between the rs144848 polymorphism and cancer risk. Literature was searched from the databases of PubMed, Embase and Google Scholar before April 2016. The fixed or random effects model was used to calculate pooled odd ratios on the basis of heterogeneity. Meta-regression, sensitivity analysis, subgroup analysis and publication bias assessment were also performed using STATA 11.0 software according to Preferred Reporting Items for Systematic Reviews and Meta-Analyses 2009. A total of 40 relevant studies from 30 publications including 34,911 cases and 48,329 controls were included in the final meta-analysis. Among them, 22 studies focused on breast cancer, seven on ovarian cancer, five on non-Hodgkin lymphoma, and the remaining six studies examined various other cancers. The meta-analysis results showed that there were significant associations between the rs144848 polymorphism and cancer risk in all genetic models. Stratified by cancer type, the rs144848 polymorphism was associated with non-Hodgkin lymphoma. Stratified by study design, the allele model was associated with breast cancer risk in population-based studies. The meta-analysis suggests that the *BRCA2* rs144848 polymorphism may play a role in cancer risk. Further well-designed studies are warranted to confirm these results.

## INTRODUCTION

Cancer is one of the most common diseases causing considerable morbidity and mortality worldwide. Environmental and genetic factors together contribute to the development of cancers [1–4]. It has been reported that DNA damage and repair is an important factor in carcinogenesis [5–7]. *BRCA2* is a well-known cancer susceptibility gene involved in the repair of double-stranded DNA breaks which functions by regulating the intracellular shuttling and activity of RAD51, another critical protein in homologous recombination [8–10]. Studies have shown that cancer carcinogenesis is related to abnormalities in DNA repair mechanisms partially caused by a change in gene function which can result from genetic polymorphisms [11, 12].

Within the last few years, many studies have focused on the association between *BRCA2* gene polymorphisms and cancer risk, including breast cancer, ovarian cancer, non-Hodgkin lymphoma, prostate cancer and others [13–18]. The rs144848 is the only common non-synonymous polymorphism in exon 10 of the *BRCA2* gene [19]. The change from A to C in the rs144848 polymorphism results in an asparagine-to-histidine transition (N372H) which may affect BRCA2 structure at residues 290-453, a region which has been determined to interact with the histone acetyltransferase P/CAF prior to transcriptional activation of target genes [20]. Over the past decade, many association studies have been conducted to explore the role of the rs144848 N372H polymorphism in cancer risk [13, 15, 17, 18, 21–40], but it is still inconclusive whether this polymorphism in the *BRCA2* gene is associated with susceptibility to cancer. Therefore, we performed a systematic review and meta-analysis of published studies focused on the association between the rs144848 polymorphism and cancer risk. Our in-depth analysis may drive a more precise estimation of risk which could in turn help identify additional genetic targets for future therapeutic interventions.

## RESULTS

### Study characteristics

**Figure 1:**
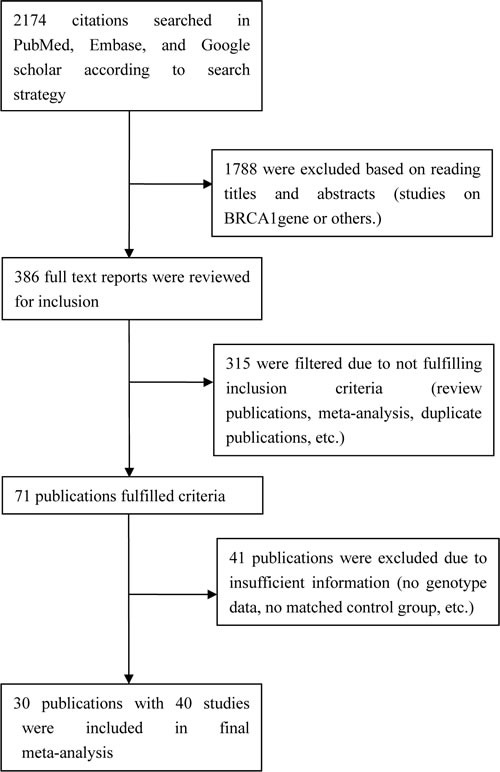
Study flow diagram

A flow diagram for the search strategy is shown in Figure 1. Based on the search strategy, 2,174 articles were identified in the initial search. After reading titles and abstracts, 1,788 articles were excluded and 386 articles were reviewed for full text. According to the study inclusion/exclusion criteria, 40 relevant studies from 30 publications including 34,911 cases and 48,329 controls were used for the final meta-analysis [13–15, 17, 18, 21, 23–40, 46–52]. Nine studies were medium quality and 31 studies were high quality. The main characteristics of these included studies are shown in Table 1.

**Table 1:** Characteristics of included studies that contributed to associations between rs144848 and cancer risk

Study [ref] per SNP	Year	Race/ethnicity	Source^a^	Cases				Controls				Allele frequencies		NOS assessment	Cancer type
				Total	NN	NH	HH	Total	NN	NH	HH	Cases^b^	Controls^b^		
Healey et al. [12]	2000	Caucasian	PB	234	116	99	19	266	138	115	13	0.71	0.73	7	Breast
Healey et al. [12]	2000	Caucasian	PB	1667	858	664	145	1201	631	493	77	0.71	0.73	7	Breast
Healey et al. [12]	2000	Caucasian	PB	450	236	180	34	228	124	94	10	0.72	0.75	7	Breast
Healey et al. [12]	2000	Caucasian	PB	659	325	285	49	866	433	373	60	0.71	0.72	7	Breast
Healey et al. [12]	2000	Caucasian	PB	449	270	154	25	453	277	152	24	0.77	0.78	7	Breast
Spurdle et al. [45]	2002	Caucasian	PB	1397	720	548	129	775	417	308	50	0.71	0.74	7	Breast
Ishitobi et al. [22]	2003	Asian	HB	149	97	47	5	144	85	56	3	0.81	0.78	7	Breast
Menzel et al. [24]	2004	Caucasian	PB	211	104	91	16	912	482	361	69	0.71	0.73	7	Breast
Menzel et al. [24]	2004	Caucasian	PB	94	53	35	6	152	84	57	11	0.75	0.74	7	Breast
Cox et al. [44]	2005	Caucasian	Nested	1285	695	501	89	1660	884	647	129	0.74	0.73	7	Breast
Millikan et al. [25]	2005	African	PB	762	564	183	15	675	510	153	12	0.86	0.87	7	Breast
Millikan et al. [25]	2005	Caucasian	PB	1265	662	521	82	1135	579	467	89	0.73	0.72	7	Breast
Garcia-Closas et al. [21]	2006	Caucasian	PB	3161	1617	1278	266	2701	1412	1057	232	0.71	0.72	7	Breast
Garcia-Closas et al. [21]	2006	Caucasian	PB	1968	1007	826	135	2276	1239	897	140	0.72	0.74	7	Breast
Johnson et al. [47]	2007	Caucasian	NA	473	233	201	39	2461	1278	993	190	0.71	0.72	6	Breast
Palli et al. [48]	2007	Caucasian	PB	91	48	31	12	261	127	107	27	0.70	0.69	6	Breast
Baynes et al. [46]	2007	Caucasian	PB	4537	2306	1892	339	4339	2182	1824	333	0.72	0.71	7	Breast
Seymour et al. [49]	2008	Caucasian	HB	252	127	111	14	100	50	44	6	0.72	0.72	6	Breast
Dombernowsky et al. [19]	2009	Caucasian	PB	1200	604	503	93	4119	2129	1677	313	0.71	0.72	6	Breast
Juwle et al. [23]	2012	Asian	NA	100	68	28	4	50	39	8	3	0.82	0.86	6	Breast
Hasan et al. [11]	2013	African	HB	100	38	33	29	100	33	32	35	0.55	0.49	6	Breast
Jumaah et al. [50]	2014	African	NA	36	26	10	0	10	10	0	0	0.86	1.00	6	Breast
Auranen et al. [26]	2003	Caucasian	PB	680	355	272	53	1546	819	629	98	0.72	0.73	7	Ovarian
Auranen et al. [26]	2003	Caucasian	PB	441	222	176	43	1097	578	445	74	0.70	0.73	7	Ovarian
Wenham et al. [28]	2003	Caucasian	PB	312	169	128	15	398	227	146	25	0.75	0.75	7	Ovarian
Beesley et al. [32]	2007	Caucasian	PB	492	249	203	40	948	502	383	63	0.71	0.73	8	Ovarian
Beesley et al. [32]	2007	Caucasian	PB	930	460	401	69	825	461	296	68	0.71	0.74	8	Ovarian
Ramus et al. [36]	2008	Mixed	Nested	4174	2196	1655	323	7402	3859	2979	564	0.72	0.72	7	Ovarian
Quaye et al. [37]	2009	Caucasian	PB	1459	779	569	111	2294	1200	925	169	0.73	0.72	7	Ovarian
Shen et al. [30]	2006	Mixed	PB	476	250	191	35	555	301	220	34	0.73	0.74	7	NHL^c^
Scott et al. [33]	2007	Caucasian	PB	757	387	307	63	676	375	253	48	0.71	0.74	7	NHL
Shen et al. [34]	2007	Caucasian	PB	556	271	236	49	498	246	203	49	0.70	0.70	7	NHL
Hill et al. [16]	2006	Mixed	PB	1116	577	441	98	926	505	361	60	0.71	0.74	7	NHL
Salagovic et al. [39]	2012	Caucasian	HB	107	62	34	11	127	82	40	5	0.74	0.80	7	NHL
Hu et al. [27]	2003	Asian	PB	120	69	39	12	231	126	95	10	0.74	0.75	6	Esophageal
Wu et al. [31]	2006	Caucasian	PB	604	306	246	52	595	332	223	40	0.71	0.75	8	Bladder
Debniak et al. [35]	2008	Caucasian	Nested	627	288	280	59	3819	1994	1580	245	0.68	0.73	6	Melanoma
Agalliu et al. [15]	2010	Caucasian	PB	1269	655	498	116	1243	654	500	89	0.71	0.73	8	Prostate
Agalliu et al. [15]	2010	African	PB	142	104	36	2	79	59	18	2	0.86	0.86	8	Prostate
Kotnis et al. [38]	2012	Asian	HB	109	35	56	18	186	81	70	35	0.58	0.62	7	Multiple

^a^ Source in control, PB population-based study, HB hospital-based study

^b^ Major allele frequency

^c^ non-Hodgkin lymphoma

### Association between *BRCA2* rs144848 polymorphism and cancer risk

**Figure 2:**
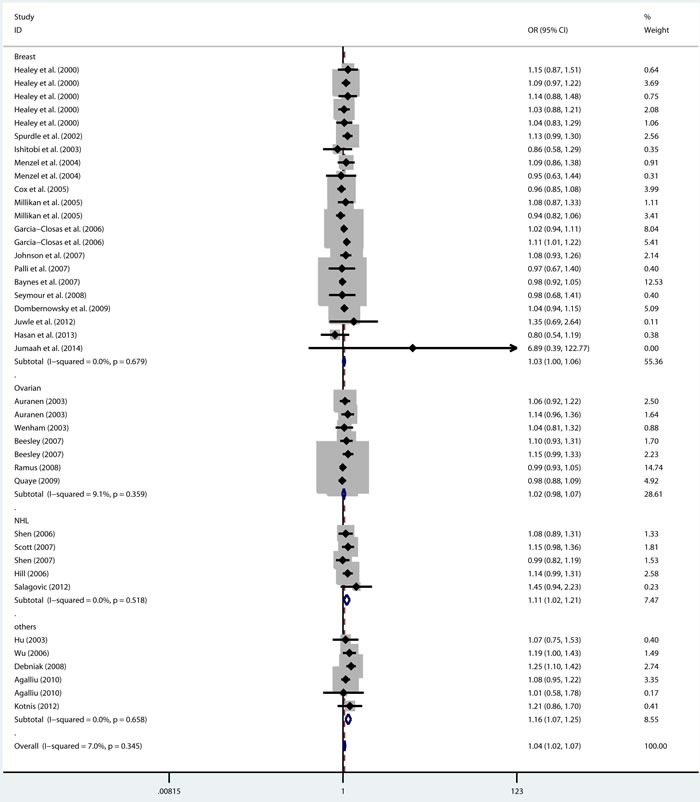
Forest plot for pooled ORs for the associations between allele model (H allele vs N allele) of rs144844 and cancer risk in the overall population Each square is proportional to the study-specific weight.

**Figure 3:**
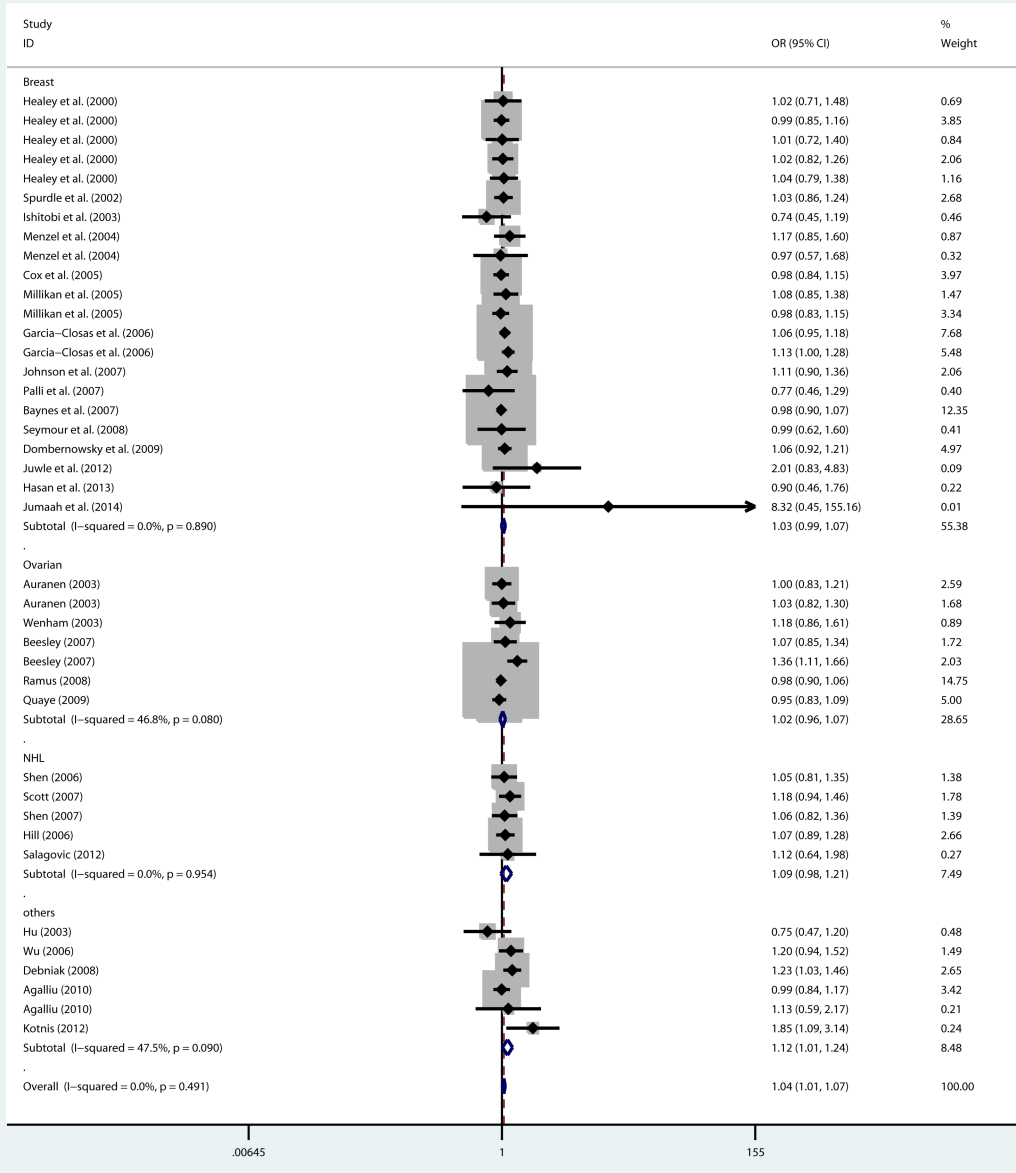
Forest plot for pooled ORs for the associations between additive model (NH vs NN) of rs144844 and cancer risk in the overall population Each square is proportional to the study-specific weight.

**Figure 4:**
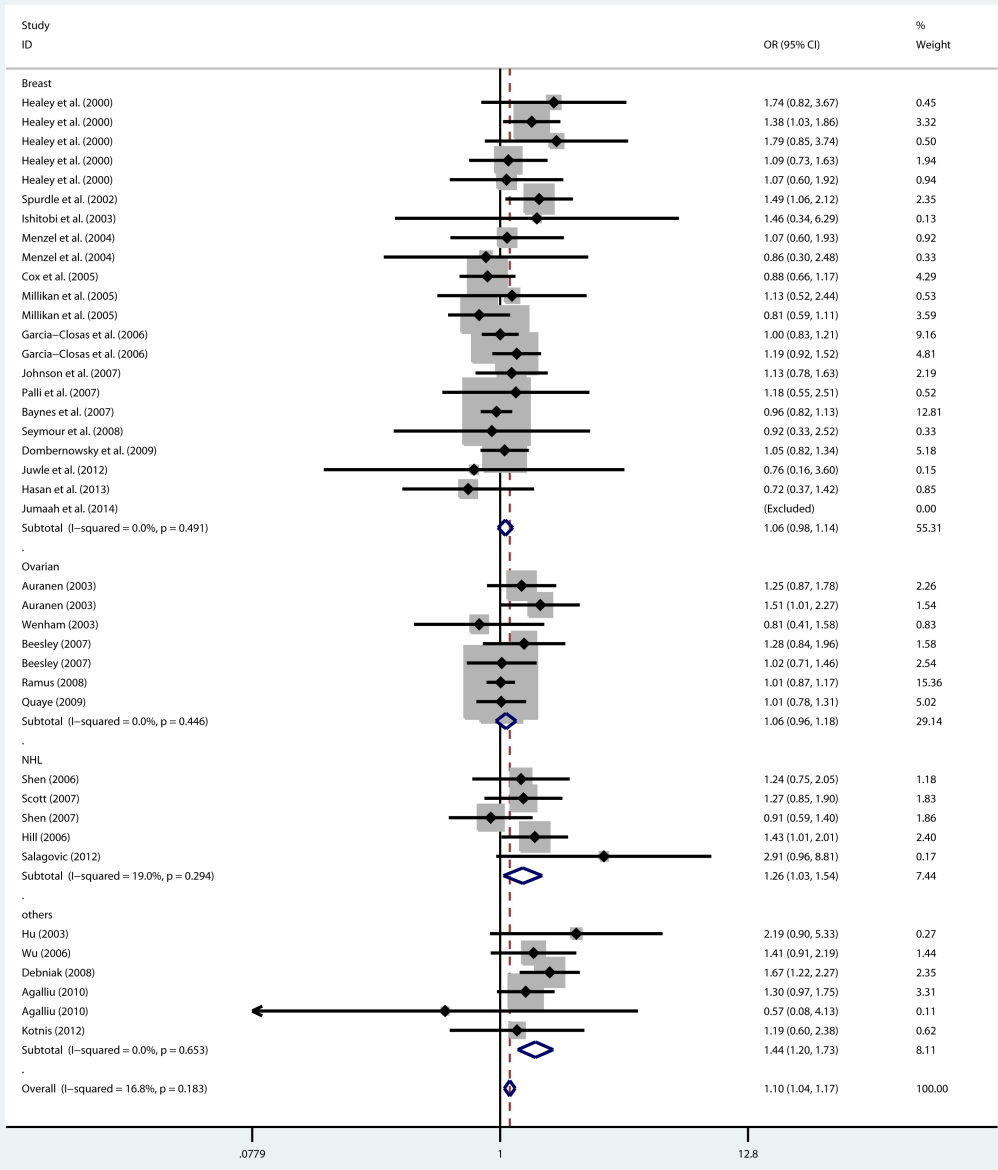
Forest plot for pooled ORs for the associations between additive model (HH vs NN) of rs144844 and cancer risk in the overall population Each square is proportional to the study-specific weight.

**Figure 5:**
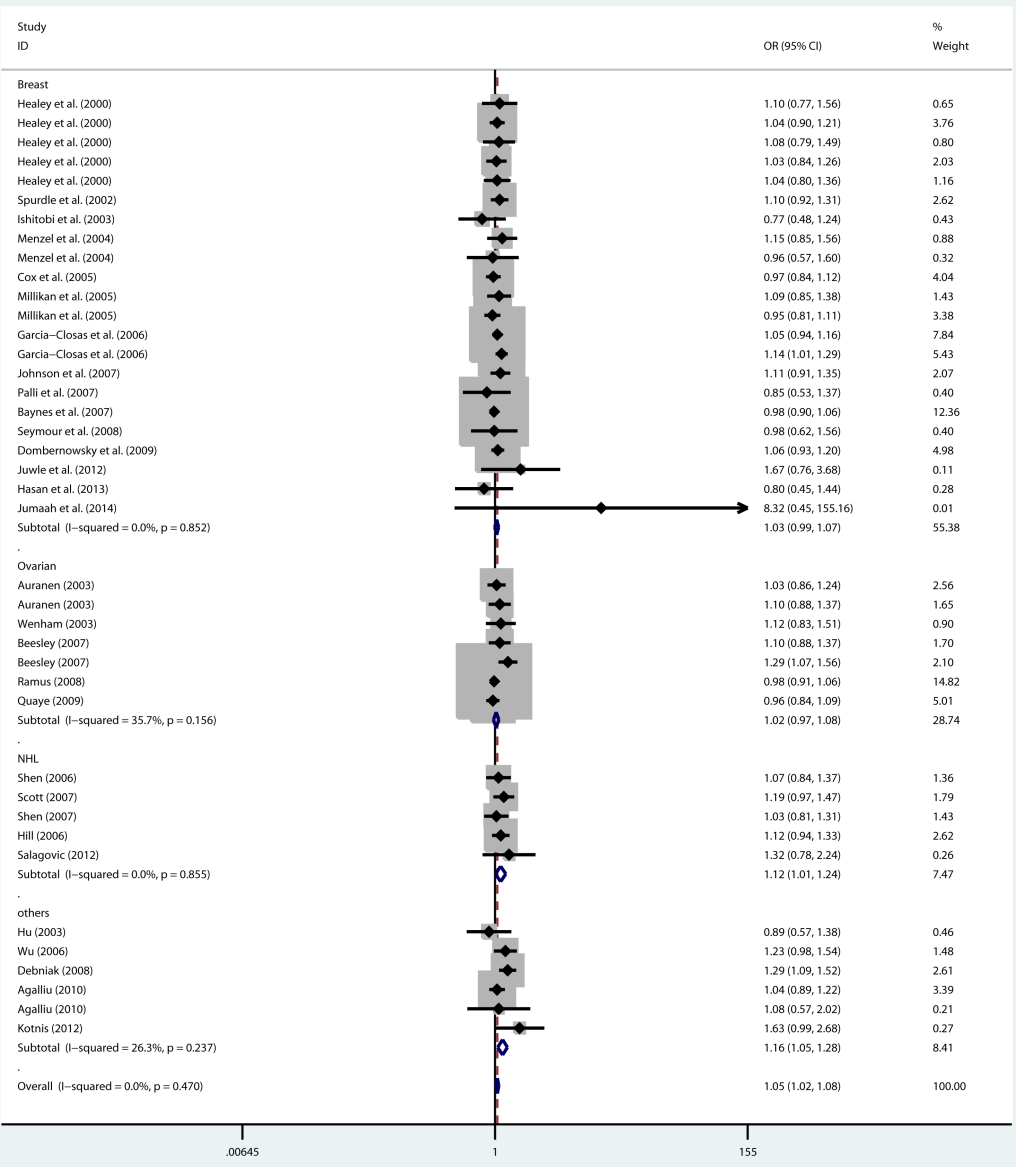
Forest plot for pooled ORs for the associations between dominant model (NH+HH vs NN) of rs144844 and cancer risk in the overall population Each square is proportional to the study-specific weight.

**Figure 6:**
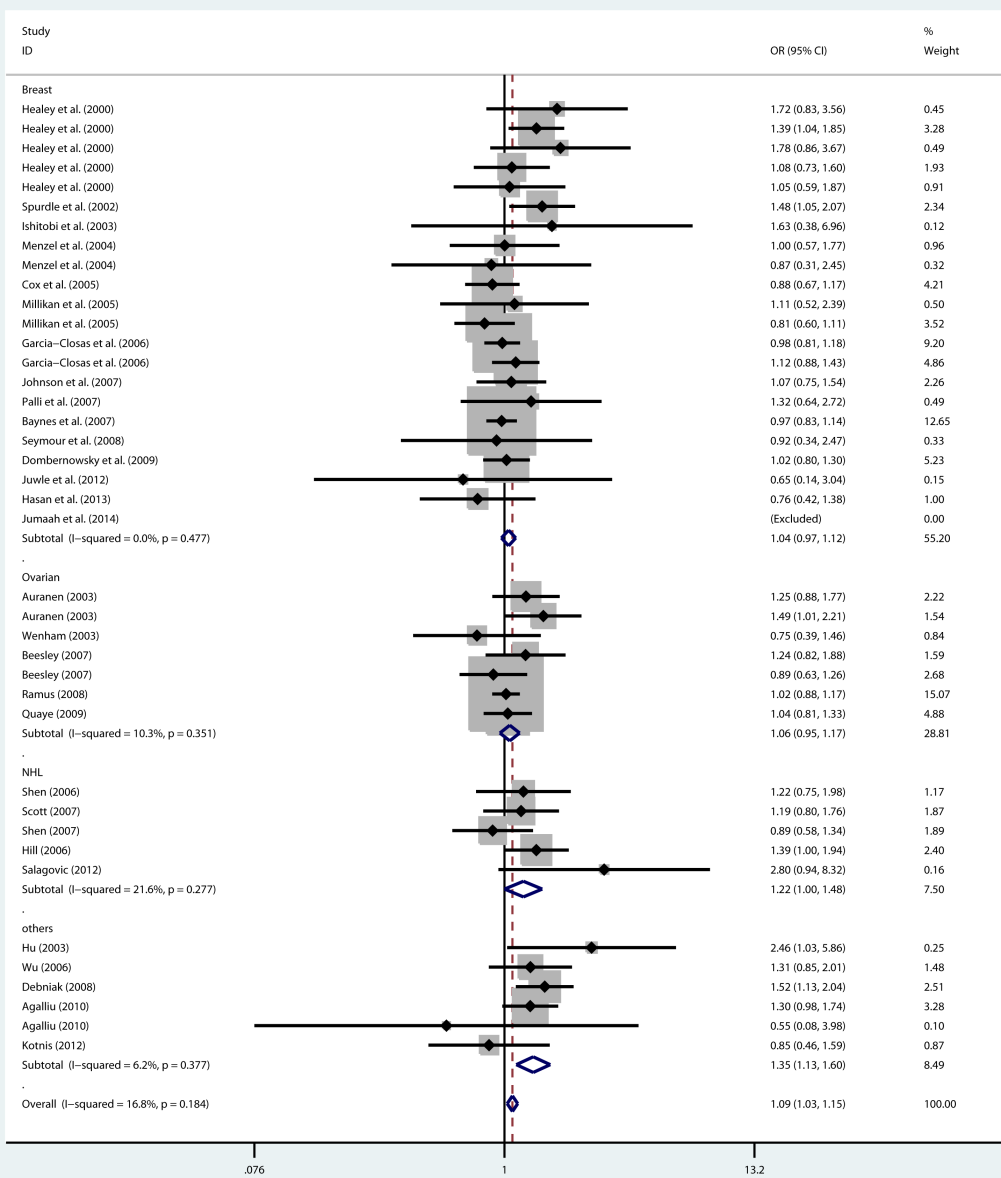
Forest plot for pooled ORs for the associations between recessive model (HH vs NH+NN) of rs144844 and cancer risk in the overall population Each square is proportional to the study-specific weight.

As shown in Table 2, there was no heterogeneity in any genetic model. The meta-analysis results showed that there were significant associations between the rs144848 polymorphism and cancer risk in all genetic models (H allele *vs*. N allele, OR = 1.044, 95% CI = 1.021-1.068, *p* < 0.001; NH *vs*. NN, OR = 1.037, 95% CI = 1.006-1.069, *p* = 0.018; HH *vs*. NN, OR = 1.104, 95% CI = 1.044-1.168, *p* = 0.001; dominant model, OR = 1.047, 95% CI = 1.018-1.078, *p* = 0.002; recessive model, OR = 1.086, 95% CI = 1.028-1.146, *p* = 0.003; Figure 2–6).

**Table 2:** Summary of OR and 95%CI for association between rs144848 polymorphism and susceptibility to cancer

Variable per SNP	*I*^2^ (%)	*p* for heterogeneity	OR (95% CI)	*p* value	*p* for publication bias	Effects model	Sensitive analysis			
							exclude	OR (95% CI)	*p* value	*p* for publication bias
H allele vs N allele	7.0	0.345	1.044 (1.021-1.068)	<0.001^a^	0.045	fixed	[36]	1.053 (1.028-1.080)	<0.001^a^	0.143
NH vs NN	0.0	0.491	1.037 (1.006-1.069)	0.018^a^	0.147	fixed	[36]	1.048 (1.014-1.082)	0.005^a^	0.352
HH vs NN	16.8	0.183	1.104 (1.044-1.168)	0.001^a^	0.066	fixed	[46]	1.125 (1.060-1.194)	<0.001^a^	0.148
Dominant model	0.0	0.470	1.047 (1.018-1.078)	0.002^a^	0.069	fixed	[36]	1.059 (1.026-1.092)	<0.001^a^	0.069
Recessive model	16.8	0.184	1.086 (1.028-1.146)	0.003^a^	0.114	fixed	[46]	1.102 (1.040-1.168)	0.001^a^	0.214

^a^ Statistically significant

### Meta-regression analysis

The following covariates were considered for meta-regression: ethnicity, study design and cancer type. The results showed that cancer type contributed to effect in the meta-analysis (H allele *vs*. N allele, *p* = 0.011; HH *vs*. NN, *p* = 0.006; dominant model, *p* = 0.039; recessive model, *p* = 0.011).

### Subgroup analysis by cancer type stratification

Based on cancer type, four groups were included in the meta-analysis: breast cancer group, ovarian cancer group, non-Hodgkin lymphoma group and other cancers group. The results showed that the rs144848 polymorphism was not associated with breast cancer or ovarian cancer in any model. However, the rs144848 polymorphism was associated with non-Hodgkin lymphoma in four models (H allele *vs*. N allele, OR = 1.110, 95% CI = 1.023-1.205, *p* = 0.012; HH *vs*. NN, OR = 1.263, 95% CI = 1.035-1.542, *p* = 0.022; dominant model, OR = 1.118, 95% CI = 1.008-1.240, *p* = 0.035; recessive model, OR = 1.216, 95% CI = 1.002-1.476, *p* = 0.048) and with other cancers in all genetic models (Table 3).

**Table 3:** Summary of OR and 95% CI for association of rs144848 polymorphism with cancer risk by cancer type stratification

Subgroup	*p* for heterogeneity	*I*^2^ (%)	OR (95% CI)	*p* value	Effects model
N allele vs H allele					
Breast cancer	0.679	0.0	1.028 (0.997-1.060)	0.075	fixed
Ovarian cancer	0.359	9.1	1.024 (0.981-1.068)	0.280	fixed
NHL	0.518	0.0	1.110 (1.023-1.205)	0.012^a^	fixed
Others	0.658	0.0	1.158 (1.074-1.249)	<0.001^a^	fixed
NH vs NN					
Breast cancer	0.890	0.0	1.029 (0.988-1.072)	0.166	fixed
Ovarian cancer	0.080	46.8	1.015 (0.959-1.074)	0.604	fixed
NHL	0.954	0.0	1.090 (0.977-1.215)	0.122	fixed
Others	0.090	47.5	1.117 (1.009-1.236)	0.033^a^	fixed
HH vs NN					
Breast cancer	0.491	0.0	1.056 (0.978-1.139)	0.162	fixed
Ovarian cancer	0.446	0.0	1.063 (0.957-1.180)	0.253	fixed
NHL	0.294	19.0	1.263 (1.035-1.542)	0.022^a^	fixed
Others	0.653	0.0	1.439 (1.199-1.726)	<0.001^a^	fixed
Dominant model					
Breast cancer	0.852	0.0	1.033 (0.994-1.074)	0.097	fixed
Ovarian cancer	0.156	35.7	1.022 (0.969-1.079)	0.420	fixed
NHL	0.855	0.0	1.118 (1.008-1.240)	0.035^a^	fixed
Others	0.237	26.3	1.162 (1.055-1.280)	0.002^a^	fixed
Recessive model					
Breast cancer	0.477	0.0	1.044 (0.969-1.124)	0.259	fixed
Ovarian cancer	0.351	10.3	1.057 (0.954-1.170)	0.290	fixed
NHL	0.277	21.6	1.216 (1.002-1.476)	0.048^a^	fixed
Others	0.377	6.2	1.346 (1.130-1.603)	0.001^a^	fixed

^a^ Statistically significant

### Association between *BRCA2* rs144848 polymorphism and breast cancer risk

There were 22 breast cancer studies with different ethnicities and study designs. To assess the role of genetic background and the source of the control population in breast cancer risk, we carried out a subgroup analysis. In the analysis of genetic background, the overall population was divided into three subgroups, Caucasian, Asian, and African. The results showed that no statistically significant association was observed in any population (Table 4). In the analysis of study design, the overall population was divided into two subgroups, population-based studies and hospital-based studies. The results showed that the allele model was associated with the risk of breast cancer based on population-based studies (H allele *vs*. N allele, OR = 1.034, 95% CI = 1.000-1.068, *p* = 0.047; Table 5).

**Table 4:** Summary of OR and 95% CI for association of rs144848 polymorphism with breast cancer risk by ethnicity stratification

Subgroup	*p* for heterogeneity	*I*^2^ (%)	OR (95% CI)	*p* value	Effects model
N allele vs H allele					
Caucasian	0.690	0.0	1.029 (0.997-1.061)	0.075	fixed
Asian	0.262	20.5	0.974 (0.692-1.372)	0.882	fixed
African	0.185	40.8	1.024 (0.850-1.235)	0.801	fixed
NH vs NN					
Caucasian	0.970	0.0	1.028 (0.986-1.072)	0.189	random
Asian	0.050	74.0	1.133 (0.427-3.006)	0.801	random
African	0.337	8.1	1.069 (0.798-1.430)	0.656	random
HH vs NN					
Caucasian	0.332	10.4	1.060 (0.981-1.146)	0.138	fixed
Asian	0.551	0.0	1.086 (0.377-3.124)	0.879	fixed
African	0.388	0.0	0.877 (0.529-1.455)	0.612	fixed
Dominant model					
Caucasian	0.925	0.0	1.033 (0.993-1.075)	0.106	fixed
Asian	0.101	62.8	0.955 (0.640-1.424)	0.820	fixed
African	0.244	29.2	1.065 (0.855-1.325)	0.575	fixed
Recessive model					
Caucasian	0.333	10.3	1.048 (0.972-1.130)	0.220	fixed
Asian	0.395	0.0	1.078 (0.378-3.072)	0.888	fixed
African	0.443	0.0	0.876 (0.548-1.399)	0.579	fixed

**Table 5:** Summary of OR and 95% CI for association of rs144848 polymorphism with breast cancer risk by the study design stratification

Subgroup	*p* for heterogeneity	*I*^2^ (%)	OR (95% CI)	*p* value	Effects model
H allele vs N allele					
PB	0.691	0.0	1.034 (1.000-1.068)	0.047^a^	fixed
HB	0.759	0.0	0.883 (0.707-1.103)	0.273	fixed
Others	0.264	24.5	1.011 (0.923-1.108)	0.810	fixed
NH vs NN					
PB	0.953	0.0	1.030 (0.986-1.076)	0.182	fixed
HB	0.684	0.0	0.864 (0.638-1.171)	0.346	fixed
Others	0.174	39.6	1.050 (0.930-1.186)	0.428	fixed
HH vs NN					
PB	0.315	12.4	1.076 (0.991-1.168)	0.082	fixed
HB	0.677	0.0	0.844 (0.501-1.422)	0.525	fixed
Others	0.559	0.0	0.957 (0.763-1.200)	0.702	fixed
Dominant model					
PB	0.916	0.0	1.037 (0.995-1.081)	0.085	fixed
HB	0.750	0.0	0.856 (0.642-1.141)	0.290	fixed
Others	0.195	36.2	1.035 (0.922-1.162)	0.558	fixed
Recessive model					
PB	0.297	14.0	1.063 (0.982-1.151)	0.132	fixed
HB	0.625	0.0	0.867 (0.538-1.398)	0.558	fixed
Others	0.627	0.0	0.943 (0.757-1.175)	0.600	fixed

^a^ Statistically significant

### Sensitivity analysis

To determine the degree to which an individual study affected the overall OR estimates, one-way sensitivity analysis was performed by excluding one study at a time and sequentially recalculating the overall effect. The results showed no influence on pooled ORs and 95% CIs as individual studies were excluded.

### Publication bias

Publication bias was observed in only one model (H allele *vs*. N allele, *p* = 0.045; Table 2). However, there was no significant publication bias in any genetic model (*p* > 0.05) after sensitivity analysis. Trim and fill results showed that the adjusted risk estimate remained significant (H allele *vs*. N allele, OR = 1.028, 95% CI = 1.006-1.050, *p* = 0.014), which confirmed that the results of this meta-analysis were statistically robust.

## DISCUSSION

The mechanisms underlying carcinogenesis are still not fully clear, but it has been suggested that genetic and environmental factors play the most important role in the development of cancer. The BRCA2 protein can regulate homologous recombination by interacting with the RAD51 recombinase, and many studies have suggested that the rs144848 polymorphism in the *BRCA2* gene is a susceptibility locus for cancers [8]. However, until now, there has been no consistent result regarding the association between the rs144848 N372H polymorphism and cancer risk. To explain these contradictory results, a meta-analysis including 34,911 cases and 48,329 controls was conducted and five genetic models were utilized to assess the association between the *BRCA2* rs144848 polymorphism and the risk of cancer.

In our meta-analysis, the results showed that there was no heterogeneity in any genetic model in overall population, while associations were observed between the rs144848 polymorphism and cancer risk in all genetic models. Meta-regression analysis suggested that ethnicity and study design had no influence on overall effect, but cancer type did contribute to effect (H allele *vs*. N allele, *p* = 0.011; HH *vs*. NN, *p* = 0.006; dominant model, *p* = 0.039; recessive model, *p* = 0.011). Based on cancer type, four groups were included in the meta-analysis: breast cancer group, ovarian cancer group, non-Hodgkin lymphoma group and other cancers group. The results showed that the rs144848 polymorphism was not associated with breast cancer or ovarian cancer in any model. However, the rs144848 polymorphism was associated with non-Hodgkin lymphoma in four models, and associated with other cancers in all genetic models.

The results showed a statistically significant association in all genetic models for overall population. Due to the relatively large number of research studies on breast cancer, we also did a subgroup analysis in the breast cancer group. To assess the role of genetic background in breast cancer, we stratified the population by ethnicity and found no association in Caucasian, Asian, and African subgroups. Considering that the number of publications in Asian and African populations was small, we believe our results may not be reliable due to insufficient statistical power, so additional studies should be conducted to confirm our results. However, after subgroup analysis by study design stratification, we found that the *BRCA2* rs144848 N372H polymorphism was associated with increasing the risk of breast cancer in population-based studies (H allele *vs*. N allele, OR = 1.034, 95% CI = 1.000-1.068, *p* = 0.047). One-way sensitivity analysis suggested no influence of individual studies on pooled ORs and 95% CIs.

In 2006, a study from the breast cancer association consortium summarized the common breast cancer-associated polymorphisms but failed to show a significant association between the *BRCA2* rs144848 polymorphism and breast cancer [53]. In 2010, Qiu *et al*. found in a meta-analysis that the *BRCA2* rs144848 H allele may be a low-penetrant risk factor for developing breast cancer [54]. In 2014, Xue *et al*. conducted a meta-analysis to assess the association between the *BRCA2* rs144848 polymorphism and cancer susceptibility [55]. In contrast to Qiu *et al*., they did not find an association between the *BRCA2* rs144848 polymorphism and breast cancer, but did observe an association with ovarian cancer. Different results from Xue *et al*. were then obtained in 2015 by Wang *et al*., who found that the rs144848 polymorphism was not associated with ovarian cancer. Compared with this latter study, we updated and added several new studies which were strictly filtered by a quality assessment. In addition, we used five genetic models to assess the role of the *BRCA2* rs144848 polymorphism in our meta-analysis. Another important difference from Wang *et al*. was that their results were based on the risk estimates obtained without the original genotype data, whereas all studies included in our meta-analysis provided genotype data, so that our results were more precise by calculating effect directly without potential deviations and biases.

The strength of this meta-analysis is that the most current literature was included. To guarantee the quality of the meta-analysis, the Newcastle-Ottawa scale was conducted to assess the quality of included studies, and a strict procedure for data extraction was performed by two investigators according to inclusion and exclusion criteria. Furthermore, no low-quality literature was included in this meta-analysis which might possibly have influenced our results. One-way sensitivity analysis and meta-regression were also performed to increase the robustness of our conclusions. Subgroup analysis by ethnicity and the source of the control population were used to explain the effect of genetic background and study design.

There are some limitations in this meta-analysis. First, the literature search strategy was limited by language, and only published papers in English were included. Second, because we excluded literature without original data, some studies were excluded. Third, other potential interactions including environment × gene, gene × gene and some potential covariates were not considered due to insufficient information.

In conclusion, our meta-analysis determined that the *BRCA2* rs144848 polymorphism was associated with non-Hodgkin lymphoma, and indicated that the rs144848 H allele of the *BRCA2* gene may be a low-penetrate risk factor enhancing carcinogenesis in breast cancer. Further well-designed studies are warranted to clarify the mechanism and increase comprehensive understanding of the role of the *BRCA2* rs144848 polymorphism in cancer.

## MATERIALS AND METHODS

### Publication research

Studies were retrieved by searching PubMed, Embase and Google Scholar following the guidelines in Preferred Reporting Items for Systematic Reviews and Meta-Analyses (PRISMA) 2009 [41]. The last search was updated on April 2016 with the terms “cancer”, “tumor”, “BRCA”, “polymorphism”, “genetic”, “variant”, “rs144848” and “N372H”. References in potential articles were also included in order to find more relevant studies.

### Inclusion criteria

All articles were reviewed by two investigators independently. Studies were included in the meta-analysis if they met the following criteria: (1) Studies were case-control or cohort studies; (2) articles were original studies of human participants; (3) genotype distributions were available; (4) studies were published in English; and (5) articles were association studies between rs144848 polymorphism and cancer risk. If studies were drawn from the same population, only the study with the largest sample size or with a sufficient quantity of useful data was included. If an article reported the results from different studies, each study was treated as a separate comparison in our meta-analysis.

### Quality score assessment

The Newcastle-Ottawa scale was used to assess the quality of studies [42]. Three items including selection, comparability and exposure were used to calculate the score of studies with a maximum score of nine. Any disagreements were adjusted by a third reviewer. A total score of three or lower, four to six and seven or greater was considered to indicate low, medium and high quality studies, respectively.

### Data extraction

Data were extracted from included studies using a standardized form. For each study, the following information was extracted: (1) name of first author, (2) year of publication, (3) ethnicity of population, (4) source of control population and (5) sample size and genotype distribution. Ethnicity was categorized as Caucasian, Asian or African, and the study design was categorized as population-based study, hospital-based study or nested study.

### Statistical analysis

The odds ratios (ORs) with corresponding 95% confidence intervals (95% CIs) were calculated to assess the association between the rs144848 polymorphism and cancer risk. Five models were used in this meta-analysis: (1) H allele *vs*. N allele, (2) NH *vs*. NN, (3) HH *vs*. NN, (4) dominant model, (NH+HH *vs*. NN), and (5) recessive model, (HH *vs*. NH+NN). Statistical analysis was performed using STATA 11.0 (Stata Corporation, College Station, TX, USA). The chi-square test was conducted to evaluate if the studies deviated from Hardy-Weinberg equilibrium, and the threshold for disequilibrium was *p* < 0.05. Cochran's *Q* test and *I*^2^ statistic test were performed to assess heterogeneity across individual studies (*p* < 0.10 and *I*^2^ > 50% suggested heterogeneity). The fixed-effects model (the Mantel-Haenszel method) was used to estimate the pooled OR if *I*^2^ < 50%; otherwise, the random-effects model (the DerSimonian and Laird method) was used [43]. A value of *p* < 0.05 was accepted as the significance threshold for each genetic model.

Subgroup analysis was conducted based on ethnicity (Caucasian, Asian and African) and study design (population-based and hospital-based). If heterogeneity was present, meta-regression was conducted to explore the source of heterogeneity. One-way sensitivity analysis was used to assess the influence of the individual study set to the pooled ORs by sequential exclusion.

A funnel plot was performed to estimate the potential publication bias using Begg's test, in which the standard error of log (OR) was plotted against its log (OR) [44]. Egger's liner regression test was also used to evaluate publication bias with quantitative analysis as a supplement to the funnel plot [45]. The trim and fill method was used to adjust pooled ORs and 95% CIs if bias was detected.

## PRISMA CHECKLIST


